# Soluble Immune Checkpoints as Prognostic Biomarkers in Small Cell Lung Cancer Patients Treated with Chemotherapy and Anti-PD-L1

**DOI:** 10.3390/ijms27146225

**Published:** 2026-07-13

**Authors:** Albert Guinart-Cuadra, Aida Piedra, Sergio Martínez-Recio, Maria Mulet, Carlos Zamora, Rubén Osuna-Gómez, Elisabet Cantó, Maria Angels Ortiz, Andrés Barba, Judit Sanz-Beltran, Jorgina Serra-López, Luís Paz-Ares, Edurne Arriola, Alberto Luis Moreno, Rosario Garcia-Campelo, Cristina Martí-Blanco, Dolores Isla, Ángel Callejo, Margarita Majem, Silvia Vidal

**Affiliations:** 1 Group of Inflammatory Diseases, Institut de Recerca Sant Pau (IR SANT PAU), 08041 Barcelona, Spain; 2 Department of Cell Biology, Physiology and Immunology, Autonomous University of Barcelona, 08193 Bellaterra, Spain; 3 Medical Oncology Department, Hospital de la Santa Creu i Sant Pau, 08041 Barcelona, Spain; 4 Department of Medicine, Universitat Autònoma de Barcelona (UAB), 08193 Barcelona, Spain; 5 Medical Oncology Department, Hospital Universitario 12 Octubre, 28041 Madrid, Spain; 6 Medical Oncology Department, Hospital del Mar–CIBERONC, 08003 Barcelona, Spain; 7 Medical Oncology Department, Hospital Universitario Reina Sofía, 14004 Córdoba, Spain; 8 Medical Oncology Department, Complejo Hospitalario Universitario de A Coruña, 15006 A Coruña, Spain; 9 Medical Oncology Department, Hospital Universitari Sant Joan de Reus, 43204 Reus, Spain; 10 Medical Oncology Department, Hospital Clínico Universitario Lozano Blesa, 50009 Zaragoza, Spain; 11 AstraZeneca Farmacéutica Spain, 28033 Madrid, Spain

**Keywords:** extensive-stage small cell lung cancer, soluble immune checkpoints, prognostic biomarkers

## Abstract

Soluble immune checkpoints (sICs) have emerged as potential biomarkers in various cancers. However, their role in extensive-stage small cell lung cancer (ES-SCLC), an aggressive tumor type with limited therapeutic options and poor prognosis, remains poorly characterized. We analyzed 14 circulating sICs, leukocyte–platelet (PLT) complexes, and PD-L1 surface expression in ES-SCLC patients treated with chemoimmunotherapy (n = 41) prior to treatment and healthy donors (HD, n = 10). We assessed their associations with clinical and demographic factors and performed survival analyses using Kaplan–Meier and log-rank tests. Levels of soluble (s) PD-L1, PD-L2, BTLA, HVEM, TIM-3, and CD27 were higher in plasma from ES-SCLC patients compared to those from HD (*p* < 0.05). Patients with liver metastases exhibited higher sIC levels, and a multivariate model demonstrated discriminative power. Network analyses revealed distinct patterns of immune dysregulation between ES-SCLC and HD. We observed correlations among sICs and leukocyte–PLT complexes and leukocyte PD-L1 expression, indicating links between systemic immune status and tumor–immune interactions. Furthermore, increased concentrations of sTIM-3, sCD27, sHVEM and sPD-L1 were associated with a poor prognosis. Overall, sICs reflect relevant clinical, prognostic, and immunological features in ES-SCLC. Their plasma measurement could aid in non-invasive patient stratification regarding liver metastases and survival risk.

## 1. Introduction

Small cell lung cancer (SCLC) is a neuroendocrine subtype of lung cancer that accounts for 15% of all lung cancers, with smoking as the primary risk factor [1]. It is the most aggressive form of lung cancer, and more than two-thirds of all cases are diagnosed in the extensive stage (ES) [2]. Clinical trial screening strategies have not led to significant improvements in disease detection nor survival, as the rapid growth of the tumor often results in its emergence during the intervals between computed tomography scans [3]. Platinum-based chemotherapy has been the standard of care in SCLC for decades. Although SCLC patients rapidly respond to chemotherapy, this response is not durable due to treatment resistance, resulting in an overall survival (OS) < 14 months and a 2-year survival rate < 10% [4]. The therapeutic landscape has recently evolved with the introduction of novel treatment modalities, among which chemotherapy combined with immunotherapy has shown particularly encouraging potential. This is supported by the CASPIAN and IMpower133 clinical trials, which demonstrated a significant improvement with the addition of programmed death ligand 1 (PD-L1) inhibitors to platinum–etoposide chemotherapy [5,6].

Although immunotherapy has been integrated into the therapeutic landscape of SCLC, the majority of patients fail to benefit and may still be exposed to immune-related toxicities [6]. Not only are these patients at risk of potentially harmful side effects, but the substantial economic burden associated with these treatments for both individuals and healthcare systems must also be considered when selecting appropriate candidates [7]. Given these challenges, the urgent need to identify biomarkers capable of stratifying patients into responders and non-responders has become increasingly critical [8].

Biomarkers such as tumor mutational burden [9] and PD-L1 expression as quantified by the tumor proportion score [10,11], which are commonly used in non-small cell lung cancer (NSCLC) to assess the potential benefits of immunotherapy and patient prognosis, have also been explored in the context of SCLC. However, none of these have validated their efficacy in discriminating between responders and non-responders to treatment in SCLC, probably due to the high mutational rate [12] observed in these tumors and their low PD-L1 expression [13]. Of note, PD-L1 expression on peripheral blood cells has not yet been thoroughly evaluated and it could be an important factor as demonstrated in our previous work in NSCLC [11]. Consequently, new research efforts are focused on identifying novel biomarkers for accurate patient classification [14]. While current studies rely mainly on genetic analyses that allow the subtyping of SCLC into four groups with distinct prognoses [15] and some genes associated with patient outcomes [8,16], important gaps remain to be addressed, such as the exploration of soluble biomarkers that can be easily assessed through liquid biopsy.

In this context, another promising area of investigation is the potential role of soluble immune checkpoints (sICs) [17,18]. While these molecules have been studied in NSCLC, highlighting the importance of PD-L1, TIM-3 and LAG-3 among others [19,20], their relevance in SCLC remains underexplored, despite their potential to improve patient stratification and monitoring. Importantly, the acquisition of these biomarkers is considerably less invasive than histological methods, requiring only peripheral blood, thus enhancing patients’ quality of life and reducing hospital costs [21,22]. Although most research has focused on membrane-bound immune checkpoints expressed on tumor-infiltrating or peripheral immune cells, these approaches are often limited by sample accessibility and cellular complexity [23]. In contrast, soluble immune checkpoints released into the circulation via alternative splicing or proteolytic cleavage can function as decoy receptors, competitive ligands, or modulators of immune signaling [18]. Therefore, their analysis may complement classical tissue-based biomarkers and contribute to minimally invasive immune monitoring strategies in cancer patients.

In this study, we present measurements of various sICs in patients with ES-SCLC prior to the initiation of treatment. Our aim is to explore their potential as biomarkers for predicting treatment response and patient outcomes in ES-SCLC. Our primary hypothesis is that the baseline levels of these sICs may correlate with disease prognosis and, consequently, with overall survival.

## 2. Results

### 2.1. Patient Characteristics

We prospectively recruited 41 patients from 21 institutions, each of whom provided at least one sample, prior to initiating first-line durvalumab–platinum–etoposide. Their clinicopathological characteristics are summarized in Supplementary Table S1. The main reasons for treatment discontinuation were disease progression (59%) and death (17%), with disease progression being the leading cause of death (80%). The median follow-up for the entire cohort was 10.19 months (95% CI 8.33–12.06, IQR 6.74–21.03). As of the data cutoff, 11 patients (26.8%) were alive. Median OS was 10.3 months (95% CI 8.7–11.8), and median progression-free survival (PFS) was 7.0 months (95% CI 6.0–8.1).

### 2.2. Patients with ES-SCLC Exhibit Elevated Plasma Levels of sICs Compared to HD

**Figure 1:**
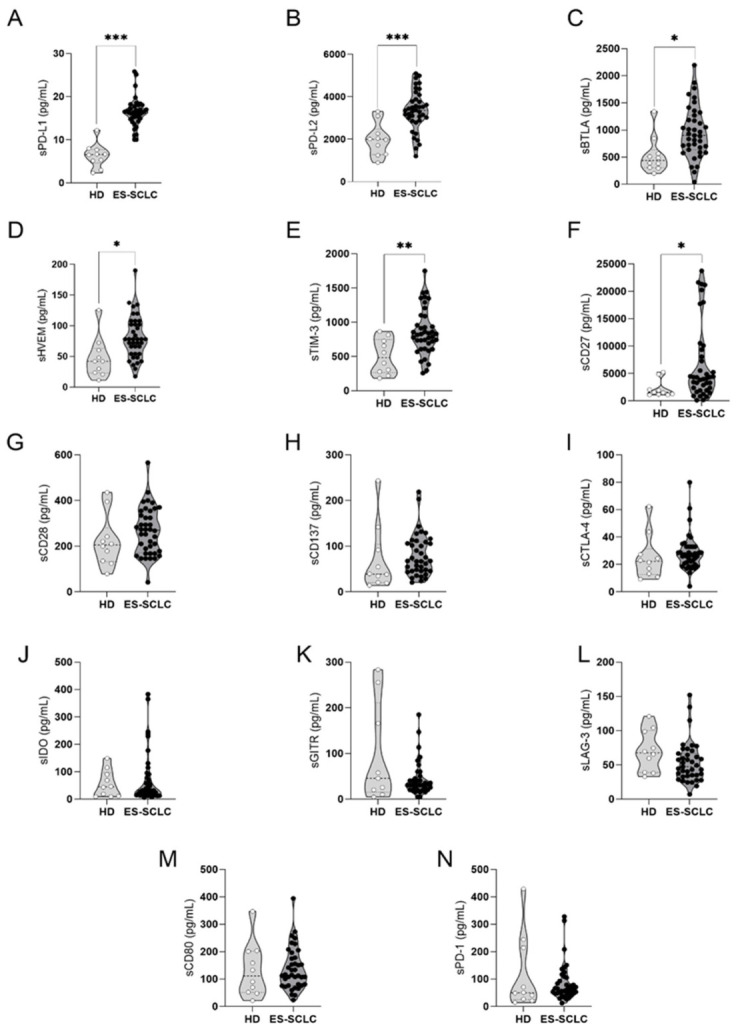
Plasma levels of sICs in ES-SCLC patients and HD. Plasma concentrations of (**A**) sPD-L1, (**B**) sPD-L2, (**C**) sBTLA, (**D**) sHVEM, (**E**) sTIM-3, (**F**) sCD27, (**G**) sCD28, (**H**) sCD137, (**I**) sCTLA-4, (**J**) sIDO, (**K**) sGITR, (**L**) sLAG-3, (**M**) sCD80, and (**N**) sPD-1 were measured in ES-SCLC patients and compared to HD. Statistical comparisons were performed using the Mann–Whitney U test or Student’s *t*-test, depending on data distribution. Significance levels are indicated as follows: * *p* < 0.05, ** *p* < 0.01, *** *p* < 0.001.

**Figure 2:**
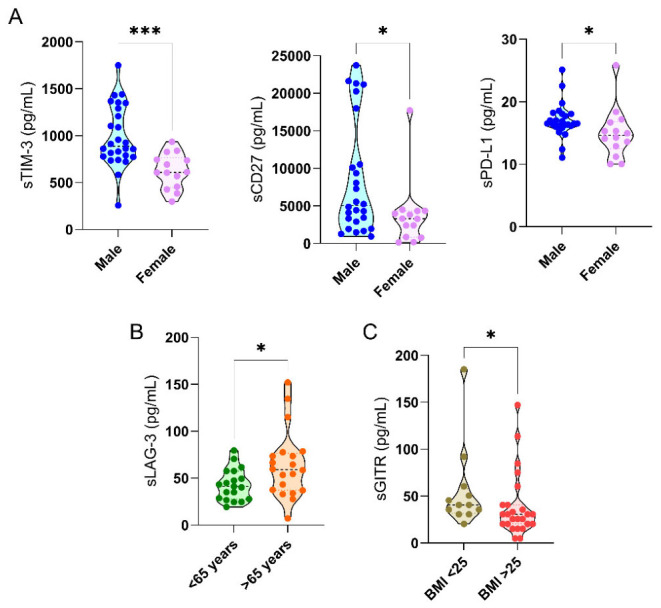
Influence of demographic factors on sICs levels in ES-SCLC. Plasma levels of sICss were analyzed according to (**A**) sex (**B**) age, and (**C**) BMI. Statistical comparisons were performed using the Mann–Whitney U test or Student’s *t*-test, depending on data distribution. Significance levels are indicated as follows: * *p* < 0.05, *** *p* < 0.001.

Plasma levels of sPD-L1, sPD-L2, sBTLA, sHVEM, sTIM-3 and sCD27 were significantly higher in the ES-SCLC group compared to the HD group (Figure 1). We next analyzed whether sIC concentrations varied within our patient cohort according to demographic factors (Figure 2). We found that males exhibited higher levels of sTIM-3, sCD27 and sPD-L1 compared to females. Patients older than 65 years exhibited higher expression of sLAG-3. Lastly, patients with a BMI ≥ 25 presented lower expression of sGITR. A complete analysis of each biomarker is available in Supplementary Table S2.

### 2.3. Elevated Levels of sTIM-3, sCD27, sHVEM, and sPD-L2 Are Associated with Liver Metastases in ES-SCLC

**Figure 3:**
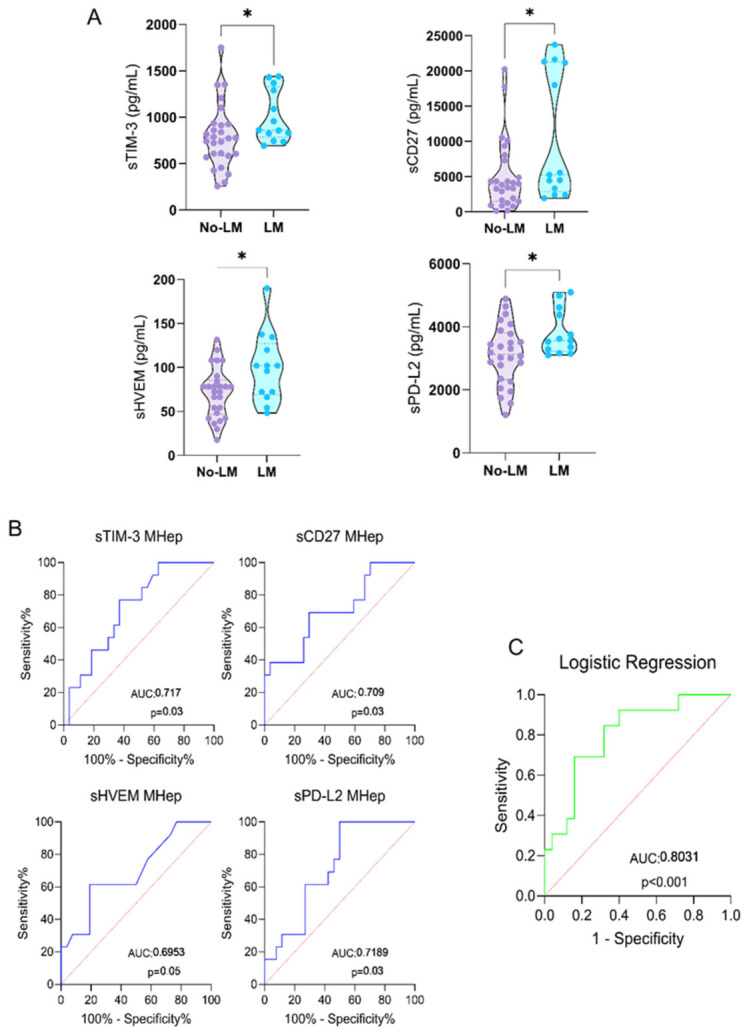
sICs as biomarkers for liver metastasis (LM) in ES-SCLC patients. (**A**) Plasma concentrations of sTIM-3, sCD27, sHVEM, and sPD-L2 in patients with and without liver metastasis. (**B**) ROC analyses evaluating the ability of these sICs to discriminate between patients with and without liver metastasis. (**C**) Multivariate ROC combining sTIM-3, sCD27, sHVEM, and sPD-L2. Significance levels are indicated as follows: * *p* < 0.05.

We observed that among the sICs elevated in the plasma of ES-SCLC patients, sTIM-3, sCD27, sHVEM, and sPD-L2 were significantly higher in patients who presented liver metastases (LMs) (Figure 3A). Detailed comparisons between the levels of all analyzed sICs and the presence of liver, CNS, and bone metastases are provided in Supplementary Table S3. The discriminative performance of sTIM-3, sCD27, sHVEM, and sPD-L2 was assessed across the entire cohort using ROC analyses to compare patients with and without LM. The area under the curve (AUC) values were 0.72 for sTIM-3, 0.71 for sCD27, 0.70 for sHVEM, and 0.72 for sPD-L2; all of these reached statistical significance (Figure 3B). We next constructed a multivariate logistic regression model incorporating the four sICs described above to assess combined predictive values. The resulting composite score demonstrated improved discriminative performance, with an AUC of 0.8 and a *p*-value < 0.001 (Figure 3C), suggesting a potential synergic effect when used in combination.

### 2.4. sICs Network Dysregulation in ES-SCLC Compared to HD

**Figure 4:**
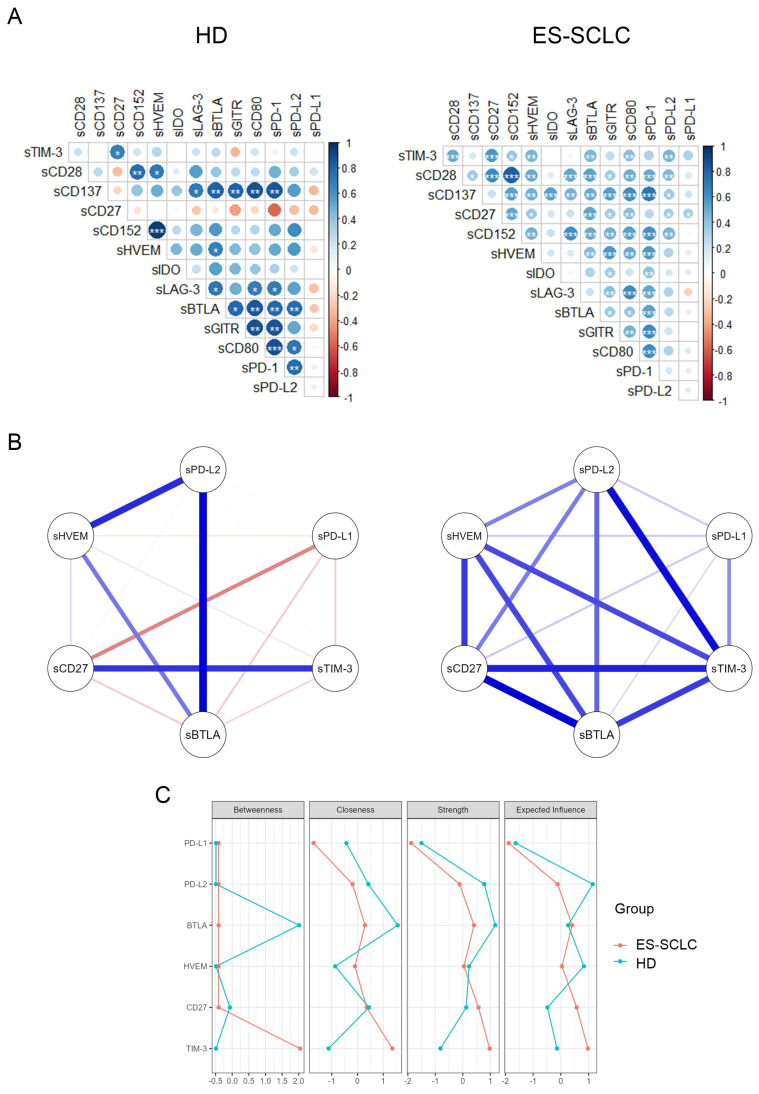
Altered network interactions among sICs in ES-SCLC patients. (**A**) Correlation matrix depicting pairwise associations between the 14 sICs in HD and ES-SCLC patients. Circle size and color intensity indicate the magnitude and direction of Spearman correlations (blue: positive, red: negative). Significance levels are indicated as follows: * *p* < 0.05, ** *p* < 0.01, *** *p* < 0.001. (**B**) Network analysis illustrating associations among sICs; blue edges denote positive correlations, and red edges negative ones. (**C**) Centrality metrics (betweenness, closeness, strength, and expected influence) highlight the relative importance of each sIC within the network. Networks were constructed using the correlation estimator.

We analyzed the interrelationships among the 14 sICs using a correlogram (Figure 4A). A distinct correlation profile was observed in ES-SCLC patients compared to HD, reflecting altered immune regulatory interactions. The ES-SCLC group exhibited a marked increase in the number of correlations, totaling 55 positive correlations. These results underscore a profound remodeling of the immune checkpoint network in ES-SCLC patients, suggesting a coordinated regulation of multiple sICs during disease. We next compared the network structure of the differentially expressed sICs between HD and ES-SCLC (Figure 4B). In HD, the network was sparse and organized in small clusters, with a limited number of strong connections particularly among sPD-L2, sHVEM, and sBTLA, or between sCD27 and sTIM-3, while sPD-L1 appeared weakly connected and showed a negative association. In contrast, the ES-SCLC network was denser, with all sICs positively connected. Notably, sPD-L1 shifted to a more central position within the network, and sTIM-3 emerged as a highly connected hub. As shown by the centrality plot (Figure 4C) of these network analyses, sTIM-3 and sBTLA presented the highest betweenness and strength in the ES-SCLC and HD groups, respectively. Globally, we found that the interplay between these molecules is disturbed in ES-SCLC.

### 2.5. PD-L1 Expression in Peripheral Immune Subsets and Its Association with sICs

**Figure 5:**
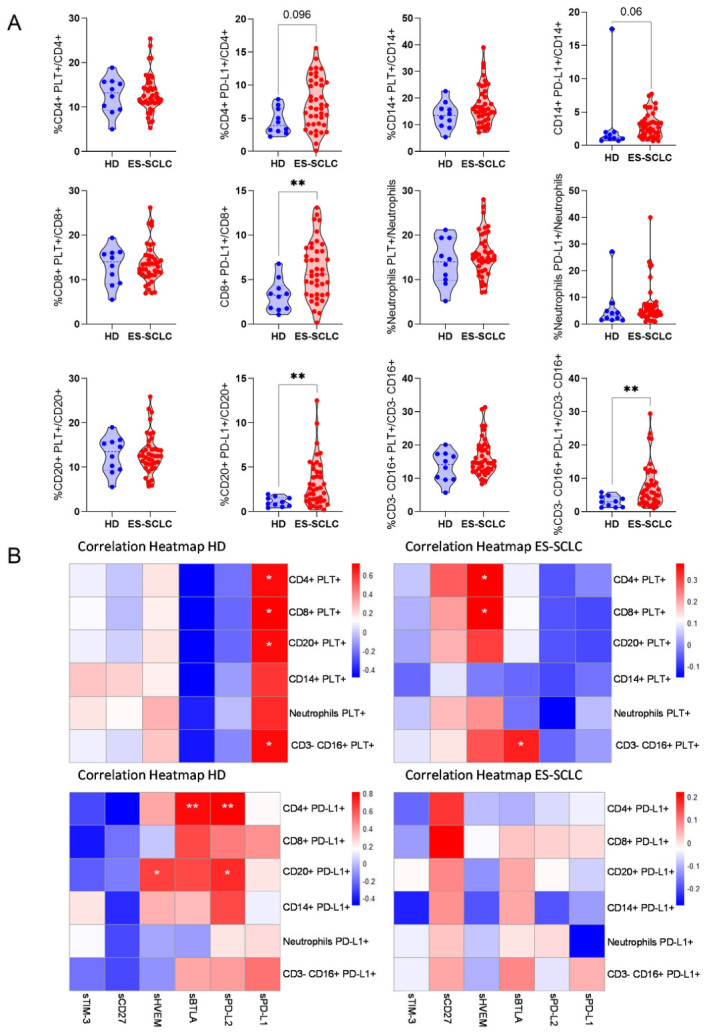
PD-L1 expression on leukocyte–PLT complexes in relation to sICs in ES-SCLC. (**A**) Percentages of CD4^+^ T cells, CD8^+^ T cells, B cells, monocytes, neutrophils, and NK cells forming complexes with PLTs, and percentages of these cells expressing PD-L1, determined by flow cytometry. Comparisons between HD and ES-SCLC patients were performed using the Mann–Whitney U test or unpaired *t*-test. (**B**) Correlation heatmap showing the associations between sICs elevated in ES-SCLC and leukocyte–PLT complexes or PD-L1 expression. Spearman or Pearson coefficients were calculated based on data distribution. Significance levels are indicated as follows: * *p* < 0.05, ** *p* < 0.01.

We analyzed the association of the expression profile of the differentially expressed sICs in ES-SCLC patients with parameters previously related to the immunobiology of NSCLC [24,25]. Specifically, we found that the frequency of leukocyte–PLT complexes did not differ significantly between HD and ES-SCLC patients. In parallel, we assessed PD-L1 expression on the surface of different peripheral blood mononuclear subpopulations. Most immune subpopulations exhibited increased PD-L1 levels in ES-SCLC patients compared to healthy donors, with the exception of neutrophils, which showed no significant difference between groups. Additionally, two subsets showed a trend towards increased expression that did not achieve statistical significance (Figure 5A).

The correlation patterns between leukocyte–PLT complexes and sICs were markedly different between the groups. In HD, positive correlations were observed between CD4+, CD8+ T cells, B cells and NK cells with PLTs and sPD-L1. In contrast, in ES-SCLC patients, correlations were found between sHVEM levels in CD4+ and CD8+ T cells, and between sBTLA and NK cells with PLTs (Figure 5B). When analyzing the correlations between PD-L1 expression on immune cells and sICs levels, the HD group displayed several positive associations: sBTLA, and sPD-L2 with PD-L1 expression on CD4+ T cells, and sHVEM and sPD-L2 with PD-L1 expression on B cells. In contrast, no correlation was found between sICs and PD-L1 expression on the different subpopulations in ES-SCLC.

### 2.6. Prognostic Value of Circulating sICs in ES-SCLC: Early Progression and Reduced Overall Survival

**Figure 6:**
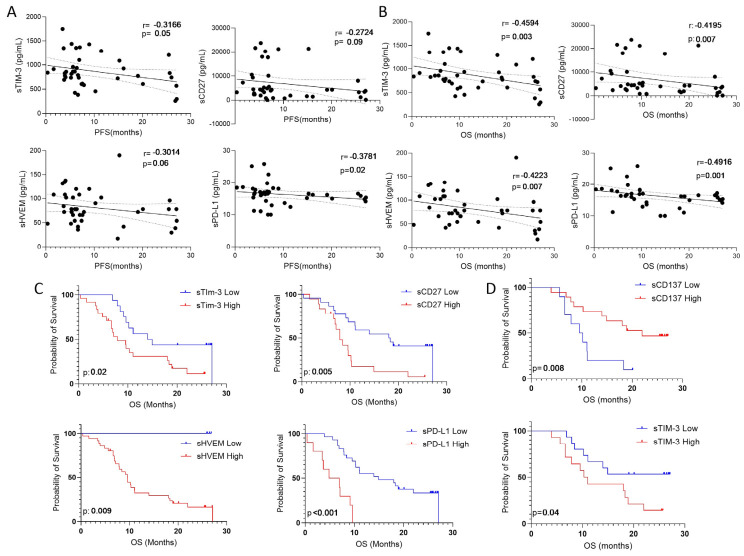
Prognostic value of sICs in ES-SCLC patients. (**A**) Correlation between progression-free survival (PFS) and plasma levels of sTIM-3, sCD27, sHVEM and sPD-L1. (**B**) Correlation between overall survival (OS) and the same sICs. Spearman or Pearson coefficients were calculated depending on data distribution. (**C**) Kaplan–Meier OS curves stratified by high vs. low plasma levels of sTIM-3, sCD27, sHVEM, and sPD-L1. Differences were assessed using the log-rank test. (**D**) sTIM-3 and sCD137 as predictors of poor prognosis in ES-SCLC patients with low levels of sPD-L1. Kaplan–Meier OS curves stratified by high vs. low plasma levels of sTIM-3 and sCD137 in the subset of ES-SCLC patients with low sPD-L1 levels. Survival differences were assessed using the log-rank test.

Exploring the clinical significance of these sICs as potential prognostic biomarkers in ES-SCLC, we found that four of the elevated sICs were associated with poor clinical outcomes. Specifically, sTIM-3 and sPD-L1 were negatively correlated with PFS while sHVEM and sCD27 showed a similar trend (Figure 6A). These associations with disease progression were further supported by OS analysis, where the same four sICs, sTIM-3, sCD27, sHVEM, and sPD-L1, also exhibited a negative correlation with survival (Figure 6B). To further validate this observation, patients were classified into two groups based on the expression levels of sTIM-3, sCD27, sHVEM, and sPD-L1. Kaplan–Meier survival analyses were then performed using the optimal cutoff points for each marker (771.96 pg/mL, 4352.21 pg/mL, 39.12 pg/mL, and 17.2 pg/mL, respectively), which were calculated in our cohort using the approach previously proposed by Cousin et al. [26]. These analyses confirmed that patients with higher levels of these sICs had a significantly shorter OS (Figure 6C), with sPD-L1 emerging as the most robust discriminator between survivors and non-survivors. Taken together, these findings suggest that elevated levels of specific sICs, particularly sTIM-3 and sPD-L1, are associated not only with earlier disease progression but also with decreased OS.

To evaluate whether the prognostic impact of these biomarkers was independent of baseline clinical characteristics, a multivariate Cox proportional hazards regression model was performed, adjusting for age, body mass index, and liver metastases. The analysis confirmed that high levels of sPD-L1 (hazard ratio [HR] = 2.57; 95% confidence interval [CI]: 1.06–6.21, *p* = 0.036) and sCD27 (HR = 2.81, 95% CI: 1.14–6.95, *p* = 0.025) remained statistically significant independent predictors of poor OS.

Within the low sPD-L1 group, some patients still experienced premature death. To investigate further this observation, we explored whether other sICs could influence the survival outcome in this group of patients. Kaplan–Meier analyses (Figure 6D) using cutoff values determined by the Youden index identified lower values of sTIM-3 (<774.48 pg/mL) and higher values of sCD137 (>47.21 pg/mL) as additional factors associated with reduced survival.

## 3. Discussion

Our study suggests that there is a distinct sICs profile in ES-SCLC patients before treatment, compared to HD. The variability in sICs levels appears to be influenced by demographic factors, and higher concentrations of specific sICs were associated with liver metastases. These findings, together with the observed links between sICs levels and survival outcomes, underscore the potential of sICs as non-invasive prognostic biomarkers for risk stratification in ES-SCLC patients treated with first-line durvalumab–platinum–etoposide.

Our investigation identified that six sICs were elevated in ES-SCLC compared to HD, specifically sPD-L1, sPD-L2, sBTLA, sHVEM, sTIM-3 and sCD27. The classification of these molecules as stimulatory or inhibitory is well defined when expressed on the cell surface; however, their soluble counterparts often exhibit less clear-cut roles. For instance, sPD-L1 [27] and sHVEM [28] have been predominantly associated with immunosuppressive effects. Conversely, sBTLA has been reported to act as an immune stimulator in certain contexts [28]. However, the immunological effects of sPD-L2, sTIM-3, and sCD27 are inconsistent or have context-dependent impacts on immune cell activation [29,30,31]. Although fewer studies have specifically focused on sICs in ES-SCLC, our results are broadly consistent with those reported in other cancer types. For example, in a cohort of advanced cancer patients including NSCLC, melanoma, SCLC, and urothelial carcinoma, higher levels of sPD-L1 showed worse survival outcomes and demonstrated that tumor PD-L1 expression did not correlate with plasma levels of sPD-L1 [19], suggesting distinct regulatory mechanisms. Similarly, a study conducted in renal cancer patients revealed elevated sTIM-3 levels were related with poorer prognosis. Additionally, sLAG-3 and sCD28 showed negative correlations with cytolytic score, which is derived from the expression of Granzyme B and Perforin [32]. These consistent findings across studies strengthen the hypothesis that sICs could provide additional clinically useful prognostic information.

Our study also indicates that demographic factors can influence circulating levels of sICs. Specifically, sTIM-3, sCD27, and sPD-L1 concentrations were found to be higher in males than in females. sLAG-3 levels were increased in older patients, and sGITR levels were lower in obese individuals. These observations align with previous studies investigating sICs molecules. In a cohort including squamous NSCLC, non-squamous NSCLC and SCLC patients, sPD-L1 levels were higher in male patients compared to females [33]. Likewise, a separate study in healthy individuals reported elevated circulating sPD-L1 levels in older versus younger participants [34].

Additional evidence from studies on membrane-bound ICs supports these patterns. Notably, older females showed reduced frequencies of CD4^+^PD-1^+^ T cells [35], highlighting a complex interplay between age and sex in shaping the immune phenotype. Although to our knowledge no studies have directly addressed the impact of BMI on sICs expression, there is evidence that this factor could modulate the response to IC inhibition in cancer [36]. Altogether, these findings suggest that demographic factors may contribute to variability in sICs expression, potentially confounding biomarker discovery in non-stratified cohorts and influencing therapeutic responses, as previously proposed in other reports [37].

Our analyses suggest that ES-SCLC patients with liver metastases exhibit higher levels of soluble TIM-3, CD27, HVEM, and PD-L2. Similar data have previously been published, showing higher levels of sTIM-3 in osteosarcoma patients with distant metastases compared to those without them [38]. Furthermore, a study also reported higher sPD-L1 levels in colorectal cancer patients with hepatic metastases [39]. Another study in rectal cancer showed that higher sCD40 levels were associated with the risk of liver metastases [40]. These differences in sICs identified as potential metastasis risk biomarkers may be attributed to the distinct biological mechanisms underlying each cancer type, although higher levels of sICs are consistently associated with a higher risk of metastasis. Our results suggest that a composite sICs-based signature could assist in the identification of ES-SCLC patients with hepatic dissemination in line with the multifactorial nature of metastatic progression [41]. Such a model could facilitate a non-invasive patient stratification, particularly considering the simplicity and feasibility of measuring sICs in peripheral blood.

Our network analyses suggest distinct patterns of interaction among sICs in ES-SCLC patients compared to HD. In contrast to HD, ES-SCLC patients exhibited a higher degree of interconnectivity, which may reflect a reconfiguration of immune regulatory networks in the disease context. Previous reports in other malignancies and autoimmune diseases have described unique interaction profiles, suggesting distinct pathophysiological mechanisms involved in autoimmune activation versus tumor-induced immune suppression and remodeling [42]. While in HD, sPD-L1 showed a positive correlation with lymphocyte–PLT complexes, this association was markedly altered in ES-SCLC. In HD, membrane PD-L1 expression correlated positively with CD4^+^ T cells and both sPD-L2 and sBTLA, and with B cells and both sPD-L2 and sHVEM. In ES-SCLC, none of the elevated sICs correlated with PD-L1 expression on immune cells. All of these observations point toward a possible decoupling of soluble and membrane checkpoint regulation in ES-SCLC, not only at the level of soluble mediators, but also in terms of cellular signaling and communication. Our data do not support a cellular origin for sPD-L1, as we found no significant correlation between sPD-L1 and its membrane-bound counterpart in the context of ES-SCLC. In contrast, a cellular origin for these soluble forms has been proposed based on observed negative correlations between sPD-1 levels and membrane-bound PD-1 expression on CD4^+^ and CD8^+^ T cells in melanoma patients [43].

In terms of survival, our results suggest that elevated sTIM-3, sCD27, sHVEM, and sPD-L1 levels were significantly associated with both shorter PFS and OS in ES-SCLC patients. These findings are consistent with previous observations in other solid malignancies where higher concentrations of sICs have also been linked to poor clinical outcomes. Several studies across lung cancer, renal cancer, and osteosarcoma have demonstrated that patients with elevated sPD-L1, sTIM-3, or sBTLA plasma levels tends to exhibit worse survival outcomes. Altogether, these data support the potential of sICs as prognostic biomarkers, which may reflect tumor-driven immune escape mechanisms [19,29,44,45]. Importantly, our findings indicate that sPD-L1 alone may not reflect the full prognostic heterogeneity in ES-SCLC. The combined assessment of sPD-L1 with sTIM-3 or sCD137 appears to enhance improves prognostic stratification, identifying biologically distinct subgroups with substantially different clinical outcomes. These findings highlight the potential clinical significance of employing stepwise combinatorial biomarker strategies to refine risk stratification in SCLC. Such approaches may aid in the development of more precise surveillance strategies, facilitating the identification of patients who, despite an initially favorable prognosis, are at risk of rapid clinical deterioration.

Although our findings provide meaningful insight, this study has several limitations. The cohort size (41 patients) is relatively small, which limits the statistical power of our survival and multivariate analyses. However, given the exclusive focus on ES-SCLC this sample is not negligible and contributes valuable data. Additionally, we assessed surface expression only for PD-L1. Extending this analysis to include the cellular expression of other sICs could enhance the mechanistic understanding and translational relevance of our observations. Furthermore, the recruitment of an independent validation cohort in future studies will be essential to corroborate our findings and strengthen their clinical relevance.

In conclusion, our exploratory results suggest that sICs may serve as non-invasive prognostic biomarkers that may aid in patient risk stratification in ES-SCLC. These preliminary findings provide a foundation for future, larger studies to validate their clinical prognostic value, while also supporting further investigation into their potential capacity as predictive biomarkers for therapeutic response.

## 4. Materials and Methods

### 4.1. Study Cohort

We collected blood samples from 41 patients with ES-SCLC enrolled in a substudy of the CANTABRICO trial (NCT04712903 Trial, EudraCT: 2020-002328-35), a phase IIIb, prospective, multicenter study which included patients with histologically confirmed ES-SCLC treated with first-line durvalumab–platinum–etoposide, who initiated treatment between February 2021 and May 2021, with January 2024 as the data cutoff. We also included blood samples from HDs (n = 10), recruited through institutional blood donation programs under standard eligibility screening criteria ensuring the absence of major acute or chronic diseases, and with an age and sex distribution comparable to that of the patient cohort. Blood samples that were used for plasma acquisition were collected in heparinized BD vacutainer tubes (BD Bioscience, Franklin Lakes, NJ, USA). Blood samples that were used for flow cytometry were recollected in EDTA BD Vacutainer tubes (BD Bioscience, Milpitas, CA, USA), previously filled with 2 mL of Transfix (Cytomark, Buckingham, UK) (stabilizing reagent) with the objective of fixing cells and maintaining the stability of cells during transportation. All patients provided written informed consent, and the study was conducted in full compliance with the ethical guidelines set forth in the Helsinki Declaration. It was approved by the Research Ethics Board of Hospital de la Santa Creu i Sant Pau. Patient data were obtained from electronic health records acquired by each institution.

### 4.2. Determination of sICs Concentrations in Plasma of ES-SCLC Patients and HD

Plasma levels of sCD27, sCD28, sCD137, sGITR, sHVEM, sBTLA, sCD80, sCTLA-4, sIDO, sLAG-3, sPD-1, sPD-L2 and sTIM-3 were determined by Luminex (Thermo Fisher, Waltham, MA, USA) and sPD-L1 was determined by ELISA (Thermo Fisher) according to the manufacturers’ instructions. The limits of detection were: 4.86 pg/mL for sCD27, 40 pg/mL for sCD28, 12 pg/mL for sCD137, 22 pg/mL for sGITR, 17 pg/mL for sHVEM, 125 pg/mL for sBTLA, 36 pg/mL for sCD80, 9.16 pg/mL for sCTLA-4, 2.93 pg/mL for sIDO, 12 pg/mL for sLAG-3,6.98 pg/mL for sPD-1, 45 pg/mL for sPD-L2, 70 pg/mL for sTIM-3 and 4.7 pg/mL for sPD-L1.

### 4.3. Whole-Blood Staining and Flow Cytometry

EDTA whole blood (100 µL) was stained for 15 min at RT in the dark with CD3-VioGreen (REA613), CD8-Vioblue (BW135/80), CD20-APC-Vio770 (LT20), CD16-PerCP-Vio770 (REA423) (Miltenyi Biotec, Bergisch Gladbach, Germany), CD14-APC (OKT-4), CD41a-FITC (HIP8) (Immunotools, Friesoythe, Germany), and PD-L1-PE (29E.2A3) (BioLegend, San Diego, CA, USA). Samples were lysed with 2 mL of 1X BD FACS Lysing Solution for 10 min, centrifuged (300× *g*, 5 min), and resuspended in 300 µL of PBS. Singlets were selected, and platelet (PLT) aggregates were excluded using CD41a-FITC/SSC, retaining leukocyte–PLT complexes. CD4+ and CD8+ T cells were gated as CD3+CD8- and CD3+CD8+, B cells as CD3-CD20+, NK cells as CD3-CD16+, monocytes as CD14+SSC, and neutrophils as CD16+SSC, after lymphocyte gating by FSC/SSC. PD-L1 expression was assessed in each subset. PLT complexes were quantified as %CD41a+ events in each subset. Acquisition was performed on a MACSQuant Analyzer 10 (Miltenyi Biotec, Bergisch Gladbach, Germany) and analysis was performed in FlowJo v10 (FlowJo LLC, Ashland, OR, USA).

### 4.4. Statistical Analysis

Statistical analyses were performed using GraphPad Prism v10 (GraphPad Software, La Jolla, CA, USA). Data normality was assessed using the Kolmogorov–Smirnov test. Continuous variables were expressed as the mean ± SEM. Comparisons of two groups were performed using Student’s *t*-test or Mann–Whitney U, as appropriate according to the data distribution. Missing data were handled using complete-case analysis, with only subjects having available data included in each specific analysis. For multiple comparisons, *p*-values were adjusted using the Benjamini–Hochberg false discovery rate (FDR) correction. Variable networks for HD and ES-SCLC patients were constructed using JASP v0.17.2 (Amsterdam, The Netherlands) in which variables were represented as nodes and associations as edges. Network centrality was evaluated using strength, betweenness, closeness, and expected influence metrics. R v4.4.3 was used to generate correlograms (corrplot package). Receiver operating characteristic (ROC) curve analyses were performed using the pROC package, and the optimal cutoff values for continuous variables were determined in R using survminer based on maximally selected rank statistics (log-rank/Mantel–Cox method). Kaplan–Meier survival curves were generated in GraphPad Prism v10 and compared using the long-rank test. Multivariate Cox proportional hazards regression model analyses were performed using IBM SPSS Statistics v21.0 (IBM Corp., Armonk, NY, USA). Hazard ratios (HRs) are reported with 95% confidence intervals (CIs). Statistical significance was set as a two-sided *p* < 0.05.

## Data Availability

All data relevant to the study are included in the article or uploaded as Supporting Information. The datasets used and analyzed during the current study are included in the article. The data support findings of this study are available from the corresponding author upon reasonable request.

## Supplementary Materials

The following supporting information can be downloaded at: https://www.mdpi.com/article/10.3390/ijms27146225/s1.

## Abbreviations

The following abbreviations are used in this manuscript:

sICs	Soluble immune checkpoints
IC	Immune checkpoint
ES-SCLC	Extensive-stage small cell lung cancer
PLT	Platelet
HDs	Healthy donors
s	Soluble
ES	Extensive stage
SCLC	Small cell lung cancer
OS	Overall survival
PD-L1	Programmed death ligand 1
NSCLC	Non-small cell lung cancer
PFS	Progression-free survival
LMs	Liver metastases
AUC	Area under the curve

## References

[1] Rudin C.M., Brambilla E., Faivre-Finn C., Sage J. (2021). Small-cell lung cancer. Nat. Rev. Dis. Prim..

[2] Dingemans A.-M.C., Früh M., Ardizzoni A., Besse B., Faivre-Finn C., Hendriks L., Lantuejoul S., Peters S., Reguart N., Rudin C. (2021). Small-cell lung cancer: ESMO Clinical Practice Guidelines for diagnosis, treatment and follow-up 5 behalf of the ESMO Guidelines Committee. Ann. Oncol..

[3] Silva M., Galeone C., Sverzellati N., Marchianò A., Calareso G., Sestini S., La Vecchia C., Sozzi G., Pelosi G., Pastorino U. (2016). Screening with Low-Dose Computed Tomography Does Not Improve Survival of Small Cell Lung Cancer. J. Thorac. Oncol..

[4] Zugazagoitia J., Paz-Ares L. (2022). Extensive-Stage Small-Cell Lung Cancer: First-Line and Second-Line Treatment Options. J. Clin. Oncol..

[5] Horn L., Mansfield A.S., Szczęsna A., Havel L., Krzakowski M., Hochmair M.J., Huemer F., Losonczy G., Johnson M.L., Nishio M. (2018). First-Line Atezolizumab plus Chemotherapy in Extensive-Stage Small-Cell Lung Cancer. N. Engl. J. Med..

[6] Paz-Ares L., Dvorkin M., Chen Y., Reinmuth N., Hotta K., Trukhin D., Statsenko G., Hochmair M.J., Özgüroğlu M., Ji J.H. (2019). Durvalumab plus platinum–etoposide versus platinum–etoposide in first-line treatment of extensive-stage small-cell lung cancer (CASPIAN): A randomised, controlled, open-label, phase 3 trial. Lancet.

[7] Nesline M.K., Knight T., Colman S., Patel K. (2020). Economic Burden of Checkpoint Inhibitor Immunotherapy for the Treatment of Non–Small Cell Lung Cancer in US Clinical Practice. Clin. Ther..

[8] Yunchu Y., Miyanaga A., Matsuda K., Kamio K., Seike M. (2024). Exploring effective biomarkers and potential immune related gene in small cell lung cancer. Sci. Rep..

[9] Hellmann M.D., Nathanson T., Rizvi H., Mcgranahan N., Snyder A., Wolchok J.D. (2018). Genomic Features of Response to Combination Immunotherapy in Patients with Advanced Non-Small-Cell Lung Cancer. Cancer Cell.

[10] Kanan D., Kanan T., Celik S., Hacıhasanoğlu E., Oven B.B. (2024). Impact of the tumor proportion score (TPS) and the combined positive score (CPS) on the use of immunotherapy in patients with metastatic non-small cell lung cancer. J. Clin. Oncol..

[11] Zamora Atenza C., Anguera G., Riudavets Melià M., De Lamo L.A., Sullivan I., Joaquin A.B., Lopez J.S., Ortiz M.A., Mulet M., Vidal S. (2022). The integration of systemic and tumor PD-L1 as a predictive biomarker of clinical outcomes in patients with advanced NSCLC treated with PD-(L)1blockade agents. Cancer Immunol. Immunother..

[12] Denninghoff V., Russo A., de Miguel-Pérez D., Malapelle U., Benyounes A., Gittens A., Cardona A.F., Rolfo C. (2021). Small Cell Lung Cancer: State of the Art of the Molecular and Genetic Landscape and Novel Perspective. Cancers.

[13] Acheampong E., Abed A., Morici M., Bowyer S., Amanuel B., Lin W., Millward M., Gray E.S. (2020). Tumour PD-L1 Expression in Small-Cell Lung Cancer: A Systematic Review and Meta-Analysis. Cells.

[14] Xie M., Vuko M., Rodriguez-Canales J., Zimmermann J., Schick M., O’bRien C., Paz-Ares L., Goldman J.W., Garassino M.C., Gay C.M. (2024). Molecular classification and biomarkers of outcome with immunotherapy in extensive-stage small-cell lung cancer: Analyses of the CASPIAN phase 3 study. Mol. Cancer.

[15] Gay C.M., Stewart C.A., Park E.M., Diao L., Groves S.M., Heeke S., Nabet B.Y., Fujimoto J., Solis L.M., Lu W. (2021). Patterns of transcription factor programs and immune pathway activation define four major subtypes of SCLC with distinct therapeutic vulnerabilities. Cancer Cell.

[16] Xie Q., Chu H., Yi J., Yu H., Gu T., Guan Y., Liu X., Liang J., Li Y., Wang J. (2021). Identification of a prognostic immune-related signature for small cell lung cancer. Cancer Med..

[17] Chen L., Chao Y., Li W., Wu Z., Wang Q. (2024). Soluble immune checkpoint molecules in cancer risk, outcomes prediction, and therapeutic applications. Biomark. Res..

[18] Gu D., Ao X., Yang Y., Chen Z., Xu X. (2018). Soluble immune checkpoints in cancer: Production, function and biological significance. J. Immunother. Cancer.

[19] Oh S.Y., Kim S., Keam B., Kim T.M., Kim D.-W., Heo D.S. (2021). Soluble PD-L1 is a predictive and prognostic biomarker in advanced cancer patients who receive immune checkpoint blockade treatment. Sci. Rep..

[20] Hayashi H., Chamoto K., Hatae R., Kurosaki T., Togashi Y., Fukuoka K., Goto M., Chiba Y., Tomida S., Ota T. (2024). Soluble immune checkpoint factors reflect exhaustion of antitumor immunity and response to PD-1 blockade. J. Clin. Investig..

[21] Ma L., Guo H., Zhao Y., Liu Z., Wang C., Bu J., Sun T., Wei J. (2024). Liquid biopsy in cancer: Current status, challenges and future prospects. Signal Transduct. Target. Ther..

[22] Fagery M., Khorshidi H.A., Wong S.Q., Vu M., Ijzerman M. (2023). Health Economic Evidence and Modeling Challenges for Liquid Biopsy Assays in Cancer Management: A Systematic Literature Review. Pharmacoeconomics.

[23] Guo Z., Zhang R., Yang A.G., Zheng G. (2023). Diversity of immune checkpoints in cancer immunotherapy. Front Immunol..

[24] Anguera G., Mulet M., Zamora C., Osuna-Gómez R., Barba A., Sullivan I., Serra-López J., Cantó E., Vidal S., Majem M. (2024). Potential Role of Circulating PD-L1+ Leukocytes as a Predictor of Response to Anti-PD-(L)1 Therapy in NSCLC Patients. Biomedicines.

[25] Zamora C., Riudavets M., Anguera G., Alserawan L., Sullivan I., Barba A., Serra J., Ortiz M.A., Gallardo P., Perea L. (2021). Circulating leukocyte–platelet complexes as a predictive biomarker for the development of immune-related adverse events in advanced non-small cell lung cancer patients receiving anti-PD-(L)1 blocking agents. Cancer Immunol. Immunother..

[26] Cousin S., Guégan J.-P., Palmieri L.J., Metges J.P., Pernot S., Bellera C.A., Assenat E., Korakis I., Cassier P.A., Hollebecque A. (2025). Regorafenib plus avelumab in advanced gastroenteropancreatic neuroendocrine neoplasms: A phase 2 trial and correlative analysis. Nat. Cancer.

[27] Han S., Zhang Y., Yuan J., Wu Y., Zhou Y., Zhou Y., Li X., Zhou S. (2024). SPD-L1 and sPD-L2 in plasma of patients with lung cancer and their clinical significance. Cytokine.

[28] Wojciechowicz K., Spodzieja M., Wardowska A. (2024). The BTLA-HVEM complex—The future of cancer immunotherapy. Eur. J. Med. Chem..

[29] Chen C., Zhao F., Peng J., Zhao D., Xu L., Li H., Ma S., Peng X., Sheng X., Sun Y. (2024). Soluble Tim-3 serves as a tumor prognostic marker and therapeutic target for CD8+ T cell exhaustion and anti-PD-1 resistance. Cell Rep. Med..

[30] Pitts S.C., Schlom J., Donahue R.N. (2024). Soluble immune checkpoints: Implications for cancer prognosis and response to immune checkpoint therapy and conventional therapies. J. Exp. Clin. Cancer Res..

[31] Gorgulho J., Loosen S.H., Masood R., Giehren F., Pagani F., Buescher G., Kocheise L., Joerg V., Schmidt C., Schulze K. (2024). Soluble and EV-bound CD27 act as antagonistic biomarkers in patients with solid tumors undergoing immunotherapy. J. Exp. Clin. Cancer Res..

[32] Wang Q., Zhang J., Tu H., Liang D., Chang D.W., Ye Y., Wu X. (2019). Soluble immune checkpoint-related proteins as predictors of tumor recurrence, survival, and T cell phenotypes in clear cell renal cell carcinoma patients. J. Immunother. Cancer.

[33] Li R., Liang H., Shang Y., Yang Z., Wang K., Yang D., Bao J., Xi W., Zhou D., Ni W. (2025). Characteristics of Soluble PD-L1 and PD-1 Expression and Their Correlations With Immune Status and Prognosis in Advanced Lung Cancer. Asia Pac. J. Clin. Oncol..

[34] Chen Y., Wang Q., Shi B., Xu P., Hu Z., Bai L., Zhang X. (2011). Development of a sandwich ELISA for evaluating soluble PD-L1 (CD274) in human sera of different ages as well as supernatants of PD-L1+ cell lines. Cytokine.

[35] Reitsema R.D., Hid Cadena R., Nijhof S.H., Abdulahad W.H., Huitema M.G., Paap D., Brouwer E., Boots A.M.H., Heeringa P. (2020). Effect of age and sex on immune checkpoint expression and kinetics in human T cells. Immun. Ageing.

[36] Gómez-Banoy N., Ortiz E., Jiang C.S., Dagher C., Sevilla C., Girshman J., Pagano A.M., Plodkowski A.J., Zammarrelli W.A., Mueller J.J. (2024). Body mass index and adiposity influence responses to immune checkpoint inhibition in endometrial cancer. J. Clin. Investig..

[37] Ye Y., Jing Y., Li L., Mills G.B., Diao L., Liu H., Han L. (2020). Sex-associated molecular differences for cancer immunotherapy. Nat. Commun..

[38] Ge W., Li J., Fan W., Xu D., Sun S. (2017). Tim-3 as a diagnostic and prognostic biomarker of osteosarcoma. Tumor Biol..

[39] Sun J., Qiu M.Z., Mei T., Gao Y., Chang B., Zhang Y., Wang F.-H., Li S. (2020). Dynamic monitoring of serum soluble programmed cell death ligand 1 as a response predictor to chemotherapy in metastatic or recurrent gastrointestinal cancer. Transl. Cancer Res..

[40] Meltzer S., Torgunrud A., Abrahamsson H., Solbakken A.M., Flatmark K., Dueland S., Bakke K.M., Bousquet P.A., Negård A., Johansen C. (2021). The circulating soluble form of the CD40 costimulatory immune checkpoint receptor and liver metastasis risk in rectal cancer. Br. J. Cancer.

[41] Fares J., Fares M.Y., Khachfe H.H., Salhab H.A., Fares Y. (2020). Molecular principles of metastasis: A hallmark of cancer revisited. Signal Transduct. Target. Ther..

[42] Yin Q., Zhu T., Song D., Fang S., Zhou H., Guan H. (2024). Soluble Immune Checkpoints Associated With Disease Activity and Treatment Response in GD and TED. J. Clin. Endocrinol. Metab..

[43] Machiraju D., Wiecken M., Lang N., Hülsmeyer I., Roth J., Schank T.E., Eurich R., Halama N., Enk A., Hassel J.C. (2021). Soluble immune checkpoints and T-cell subsets in blood as biomarkers for resistance to immunotherapy in melanoma patients. Oncoimmunology.

[44] Kashima J., Okuma Y., Hosomi Y., Hishima T. (2019). High Serum Soluble CD27 Level Correlates with Poor Performance Status and Reduced Survival in Patients with Advanced Lung Cancer. Oncology.

[45] Gorgulho J., Roderburg C., Heymann F., Schulze-Hagen M., Beier F., Vucur M., Kather J.N., Laleh N.G., Tacke F., Brümmendorf T.H. (2021). Serum levels of soluble B and T lymphocyte attenuator predict overall survival in patients undergoing immune checkpoint inhibitor therapy for solid malignancies. Int. J. Cancer.

