# Supplementary material for: Soluble Immune Checkpoints as Prognostic Biomarkers in Small Cell Lung Cancer Patients Treated with Chemotherapy and Anti-PD-L1
Source: Int J Mol Sci. 2026 Jul 13;27(14):6225. doi: 10.3390/ijms27146225 (PMC13409894; doi:10.3390/ijms27146225)
Supplement: Supplementary file 1 [file ijms-27-06225-s001.zip › ijms-4405032-supplementary.pdf]

Supplementary table 1 (Table S1)

Patient characteristics	ES-SCLC patients
**Median age (range), years**	66 (48-84)
**Male/Female**	27/14
**White/Others**	37/4
**ECOG-PS (0/1)**	11/30
**Smoking habits**	
**Former Smoker (<100 cigarettes/life)**	20
**Current Smoker**	21
**BMI (kg/m2)**	
**<18.5**	1
**18.5-24.9**	13
**≥25**	27
**IRAEs (Yes/No)**	16/25
**CNS Metastasis (Yes/No)**	4/37
**Bone Metastasis (Yes/No)**	13/28
**LM (Yes/No)**	13/28

Abbreviations: ES-SCLC: Extensive stage small cell lung cancer; ECOG-PS: Eastern Cooperative oncology group performance status; BMI: Body mass index; CNS: Central nervous system; IRAEs: immune-related adverse effects; LM: Liver metastasis.

Supplementary table 2 (Table S2)

Subanalysis of sICs based on demographic factors										
**sICs (pg/mL)**	**Sex**		**Age (years)**		**Tobacco Status**		**BMI**		**ECOG Status**	
	**Male (n=27)**	**Female (n=14)**	**<65 (n=21)**	**≥65 (n=20)**	**Ex-smokers (n=20)**	**Smokers (n=21)**	**<25 (n=27)**	**≥25 (n=14)**	**ECOG 0 (n=11)**	**ECOG 1 (n=29)**
**sTIM-3**	991.7±328.2	623.7±186.6***	831.8±242	893.9±412	785.3±319.4	940.4±340.2	875.7±437.1	856.7±282.9	825.3±354.4	877.1±32.7
**sCD28**	275.9±88.98	247±127.4	248.8±99.63	282.8±106.7	240.9±108.7	290.7±39.83	280.4±85.26	258.8±111.8	240.1±96.68	275.6±105.7
**sCD137**	85.94±49.93	68.82±35.29	74.29±44.50	85.88±47.50	75.75±45.46	84.49±46.94	87.17±53.83	76.76±42	98.47±65.43	73.95±36.26
**CD27**	8422±7560	3729±4330*	7156±6904	6404±7115	6088±6719	7471±7241	7430±6819	6466±7090	5079±6256	7424±7166
**sCTLA-4**	30.30±12.9	25.9±13.21	24.74±7.95	32.28±16.05	27.07±12.86	29.96±13.45	27.55±6.33	28.97±15.40	24.28±8.84	30.12±14.16
**sHVEM**	82.77±37.46	77.46±30.05	76.71±29.95	85.07±39.29	79.16±34.83	82.93±35.72	90.53±30.96	76.26±36.26	84.41±27.37	17.59±79.66
**sIDO**	46.41±102.6	69.25±80.23	68.25±81.51	79.56±107.5	62.15±73.57	85.66±112.1	72.79±111.8	74.44±87.07	74.14±113.2	73.81±88.44
**sLAG-3**	50.71±27.42	59.79±34.56	42.85±16.68	64.08±35.87*	53.69±31.01	53.79±29.50	57.27±26	51.98±31.93	51.25±24.22	54.60±31.90
**sBTLA**	1057±476.5	771.6±423	1009±520	913±428.3	867.6±376.4	1052±545.2	999.6±372.3	943.2±523	838.5±539.2	1011±446.7
**sGITR**	44.74±38.52	42.86±38.15	44.57±37.59	43.81±39.20	31.83±16.70	55.88±48.07	55.62±44.70	38.69±33.78	39.87±23.54	45.78±42.26
**sCG80**	133.3±65.35	146.8±97.38	130.7±66.04	144.5±86.38	135.3±78.07	140.1±76.85	151.6±75.47	130.8±77.47	141.3±67.57	136.6±80.37
**sPD-1**	86.61±63.57	82.25±79.31	79.24±68.08	91.39±69.60	68.86±46.94	100.6±81.82	117.2±96.70	69.13±42.16*	67.57±39.98	92.07±76.20
**sPD-L2**	3503±943.8	3037±867.2	3374±672.2	3322±1147	3162±1061	6523±782.9	3018±1063	3512±836.6	3232±1199	3387±846.3
**sPD-L1**	17±2.75	14.99±3.99*	16.46±3.72	16.09±2.99	15.58±2.15	16.95±4.12	17.06±4.83	15.90±2.31	15.53±2.21	16.58±3.67

Abbreviations: sICs: soluble immune checkpoints; s: soluble; BMI: Body mass index; ECOG: Eastern Cooperative Oncology Group

Statistical comparisons between the two subgroups within each demographic variable were conducted using either the Mann–Whitney U test or the unpaired Student's t-test, based on the normality of the data distribution. *$p < 0.05$; ***$p < 0.001$ for each group vs HD.

Supplementary table 3 (Table S3)

Subanalysis of metastases presence in SCLC patients according to sICs

sICs (pg/mL)	Metastasis (Bone/Liver/CNS)		Liver Metastasis not exclusive (n bone=5. n CNS=2)		Bone Metastasis not exclusive (n liver=5. n CNS= 1)		CNS Metastasis not exclusive (n liver=2. n bone=1)	
	No (n=19)	Yes (n=22)	No (n=28)	Yes (n=13)	No (n=28)	Yes (n=13)	No (n=37)	Yes (n=4)
sTIM-3	753.7±274.1	952.2±359.2*	792.2±341	1010±279.6*	797.2±295.9	999.2±381	880.1±321.9	707.9±463.2
sCD28	256.4±135.8	273.6±68.97	253.2±116.6	292±64.43	260.8±116.2	276.3±72.41	267.1±107.3	254.3±65.56
sCD137	58.65±23.13	96.91±52.19*	70.16±42.41	100.4±47.37*	76.97±40.67	86.76±56	75.76±42.56	119.4±61.61
CD27	5462±5649	7887±7779	5017±4990	10440±8980*	6082±6464	8230±7886	6690±6773	7583±9396
sCTLA-4	28.29±17.48	28.69±8.35	26.85±14.60	31.96±8.61*	29.51±15.18	26.43±7	28.71±13.74	26.75±3.97
sHVEM	69.65±30.62	89.76±36.04	71.57±28.70	99.86±39.39*	81.71±39.17	79.56±25.42	81.07±35.77	80.40±29.72
sIDO	46.35±37.43	96.45±119.2	73.13±99.38	75.50±86.77	60.16±67.36	102.4±133.4	75.12±96.78	62.96±79.24
sLAG-3	49.58±36.16	56.96±24.30	50.34±30.11	60.54±29.27	56.97±34.41	47.29±16.91	52.48±31.23	64.79±6.924
sBTLA	864.5±471.9	1046±470.5	891.5±443.9	1121±519.6	909.3±456.8	1067±508	954.1±491	1031±322.9
sGITR	39.42±35.21	48.22±40.48	44.42±42.95	43.68±25.95	40.17±29.57	52.23±51.75	44.94±39.46	35.58±5
sCD80	97.23±51.14	169.1±78.91**	113.6±59.96	186±85.06**	130.9±78.68	151.6±72.84	1323±76.89	185.7±60.36
sPD-1	60.05±32.14	106.7±83.08*	83.03±75.55	89.95±50.36	70.86±42.96	113.8±97.73	80.30±57.79	127.7±135.5
sPD-L2	3153±847.3	3498±989.2	3114±956.7	3815±708.7*	3293±942.3	3456±946.3	3369±867.3	3155±1574
sPD-L1	15.69±2.94	16.85±3.67	16.44±3.73	15.89±2.16	15.55±2.73	17.94±4.08*	16.12±3.04	17.69±5.84

Abbreviations: sICs: soluble immune checkpoints; s: soluble: CNS: Central nervous system.
Statistical comparisons between non-metastatic and metastatic groups were made using Mann–Whitney U or unpaired t-tests, according to the distribution of each variable. *$p < 0.05$; **$p < 0.01$ for each group vs HD.
